# Clinical assessment of hepatic de novo lipogenesis in non-alcoholic fatty liver disease

**DOI:** 10.1186/s12944-016-0321-5

**Published:** 2016-09-17

**Authors:** Sabina Paglialunga, Clayton A. Dehn

**Affiliations:** 1 Global Clinical Research, Celerion, 2420 West Baseline Road, Tempe, AZ 85283 USA; 2 Current affiliation: Umbrella Corporation, San Antonio, TX USA

**Keywords:** Stable label isotope, MIDA, Fatty acid indexes, Indirect calorimetry, Fructose

## Abstract

Non-alcoholic fatty liver disease (NAFLD) is heralded as the next big global epidemic. Hepatic *de novo* lipogenesis (DNL), the synthesis of new fatty acids from non-lipid sources, is thought to play a pivotal role in the development of NAFLD. While there is currently no NAFLD-specific therapeutic agent available, pharmaceutical drugs aimed at reducing hepatic fat accretion may prove to be a powerful ally in the treatment and management of this disease. With a focus on NAFLD, the present review summarizes current techniques examining DNL from a clinical perspective, and describes the merits and limitations of three commonly used assays; stable-label isotope tracer studies, fatty acid indexes and indirect calorimetry as non-invasive measures of hepatic DNL. Finally, the application of DNL assessments in the pharmacological and nutraceutical treatment of NAFLD/NASH is summarized. In a clinical research setting, measures of DNL are an important marker in the development of anti-NAFLD treatments.

## Background

**Fig. 1:**
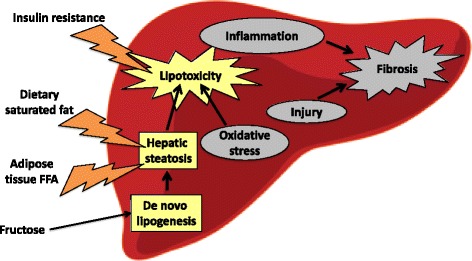
The pathophysiology of NAFLD/NASH. Excess dietary saturated fat intake as well as increased adipose tissue lipolysis contributes to fat accumulation in the liver. Overconsumption of fructose leading to upregulated *de novo* lipogenesis (DNL) also adds to hepatic steatosis. Insulin resistance and oxidative stress fuel a lipotoxic environment within the liver, which can result in hepatic inflammation and injury leading to fibrosis

In both developed and developing countries, the incidence of metabolic diseases are on the rise and are largely associated with sedentary lifestyles as well as easy access to calorie rich – nutrient low foods. Further, approximately one billion people worldwide are affected by non-alcoholic fatty liver disease (NAFLD) [1], a chronic metabolic derangement caused by an excess of lipid storage within the liver referred to as steatosis, of which obesity and type 2 diabetes are frequent comorbidities [2]. Individuals with increased liver fat content are diagnosed with NAFLD when lipid accumulation in the liver is not related to excessive alcohol intake or viral infection such as hepatitis. NAFLD is typically asymptomatic and may even be benign, but in approximately 20 % of affected individuals prolonged steatosis will lead to a severe form of the disease called non-alcoholic steatohepatitis (NASH) marked by liver inflammation and fibrosis [3]. NASH may further precipitate cirrhosis and even hepatocellular carcinoma [2]. Excessive dietary intake of saturated fatty acids, increased free-fatty acids derived from lipolysis of white adipose tissue, reduced β-oxidation within the liver and upregulated hepatic d*e novo* lipogenesis (DNL), the synthesis of new fatty acids from carbohydrates or proteins, are all thought to contribute to liver steatosis [4, 5]. Furthermore, conditions of insulin resistance and oxidative stress induce a lipotoxic environment within the liver provoking inflammation and hepatocyte injury which can lead to the development of fibrosis (Fig. 1).

There are currently no NAFLD-specific medications available on the market, and a clear pathway to approval is yet to be adopted. The unpredictable and slowly progressive nature of the condition makes the assessment of direct endpoints such as cirrhosis or mortality impractical. As such, the Food and Drug Administration (FDA) is considering the use of a surrogate outcome measures including the reversal of steatohepatitis without the progression of advanced fibrosis to examine the treatment response to novel therapies [6]. Hepatic lipid accretion is implicated in the development and progression of NAFLD (reviewed in [7, 8]), and clinical methods of examining the same are valuable signals for evaluating anti-NAFLD drug efficacy. The present review will highlight current and novel tools and techniques assessing DNL, and how this analysis is clinically applicable to evaluate the treatment of NAFLD.

## *De novo* fatty acid synthesis

**Fig. 2:**
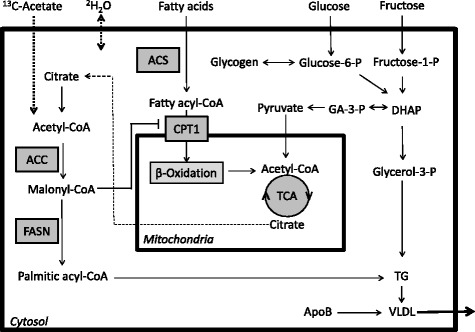
Hepatic *de novo* lipogenesis and TG secretion. The DNL process begins with the conversion of acetyl-CoA into malonyl-CoA by ACC. Malonyl-CoA is condensed with several acetyl-CoA moieties by FASN to produce a 16-carbon palmitic acyl-CoA. Three fatty acyl-CoAs are bound to a glycerol backbone to form one TG. Glucose and fructose are taken up by the hepatocyte and may be metabolized to pyruvate for energy formation in the mitochondrial or to glycerol-3-phospate for TG synthesis. Glucose can also be stored as glycogen within the liver cell. TG are combined with apoB and packaged into VLDL for secretion into the blood stream. Malonyl-CoA, also inhibits CPT1, suppressing fatty acid uptake into the mitochondria and β-oxidation. Dietary fatty acids are also taken up by the hepatocyte and converted to fatty acyl-CoA which can be oxidized or used for TG synthesis (not shown). Stable isotope labels such as ^13^C-acetate and ^2^H_2_O are used to determine the rate of DNL through the incorporation into VLDL-palmitate. ACC, acetyl-CoA carboxylase; ACS, acetyl-CoA synthase; ApoB, apolipoprotein B; CPT1, carnitine palmitoyl transferase 1; DHAP, dihydroxyacetone phosphate; FASN, fatty acid synthase; GA-3-P, glyceraldehyde-3-phosphate; P, phosphate; TCA, the citric acid cycle; TG, triglyceride; VLDL, very low density lipoprotein

**Fig. 3:**
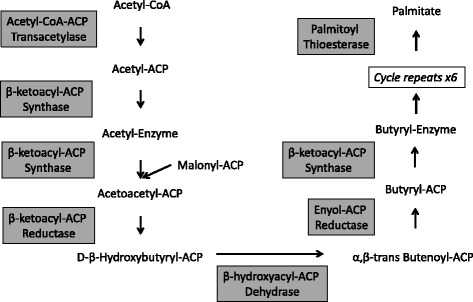
Fatty Acid Synthase Reactions. Fatty acid synthase (FASN) is a multi-complexed enzyme is responsible for the building of new fatty acids. The FASN enzymes are shown. The process begins with the condensation of malonyl-CoA onto an acyl-carrier protein group, followed by the extension of the malonyl-ACP with an acetyl-CoA subgroup. A series of reduction and dehydration steps result in the production of palmitic acid, a 16-carbon long saturated fatty acid

DNL is a highly regulated pathway, dependent upon several steps, in which key enzymes involved are upregulated in NAFLD [9, 10]. The process begins with acetyl-CoA, derived from non-lipid sources, as a building block for fatty acid synthesis. The first committed step in this pathway is the formation of malonyl-CoA from acetyl-CoA (Fig. 2). This reaction is catalyzed by acetyl-CoA carboxylase (ACC), of which there are two isoforms (ACC1 and ACC2) (reviewed in [11]). These enzymes are derived from independent genes and differ in their tissue distribution and subcellular localization. ACC1 is found in lipogenic tissue and localized in the cytosol, while ACC2 is highly expressed in skeletal and heart muscle and bound to mitochondria [12]. ACC is active in unphosphorylated states that occur during fed and insulin-stimulated conditions resulting in increasing levels of malonyl-CoA. Conversely, ACC is phosphorylated and inactivated by AMPK, interrupting the production of malonyl-CoA. Malonyl-CoA is sequestered towards DNL as a substrate required for fatty acid synthesis and it also directly regulates fatty acid oxidation as an allosteric inhibitor of carnitine palmitoyl transferase 1 (CPT1) [13, 14] (Fig. 2). CPT1 transports long-chain fatty acids into the mitochondria as the obligatory step in fatty acid oxidation. The final key regulator of DNL is fatty acid synthase (FASN). This is a multi-complexed enzyme that is responsible for the building of new fatty acids (Fig. 3). The process begins with the condensation of malonyl-CoA onto an acyl-carrier protein (ACP) group, followed by the extension of the malonyl-ACP with an acetyl-CoA subgroup. The resulting acetoacetate moiety is reduced, dehydrated, and reduced again to generate butyryl-ACP. Butyryl-ACP also under goes a round of dehydration and reduction steps and this process repeats six times to produce a 16-carbon long saturated fatty acid called palmitate. FASN is highly regulated at the transcriptional and translational level; its activity is stimulated by insulin and citrate, and inhibited by PKA, AMP and palmitoyl-CoA (review in [15]). The *de novo* fatty acids produced have multiple fates. They are used for triglyceride (TG) synthesis, oxidized through β-oxidation, and may function as intracellular signals including acting as PPAR ligands, LXR modulators, lipokines, and substrates for protein palmitoylation (reviewed in [16]).

## Hepatic DNL in health and disease

Early studies suggested hepatic DNL was not a significant in vivo contributor to fat metabolism. These initial findings were, however, likely related to the test subjects and conditions examined. In healthy lean, fasting individuals it was originally estimated that DNL contributed to <5 % of total TG synthesis [17–19]. However, under fed conditions DNL rates are ~25 % greater than fasting levels [20–24]. In addition, hepatic lipogenesis is 2-fold higher in the elderly compared to young subjects [25]. Moreover, DNL rates compared to healthy controls are upregulated approximately 5-fold in individuals with metabolic disorders such obesity [21, 24] and type 2 diabetes [23, 26]. Although the numbers of studies performed to date are limited [20, 27–29], DNL rates in NAFLD patients have reported an increase of up to 3-fold compared to BMI-matched controls [27]. Interestingly, NAFLD is more prominent in men than women [30], suggesting the potential of a protective effect of estrogen on the DNL pathway; Pramflak et al. have recently demonstrated this sexual dimorphism is related to intrahepatic fatty acid partitioning and DNL rates [31]. Augmented hepatic DNL rates are now considered a causal factor in the development of NAFLD [5, 20, 27]. Supporting this, FASN expression and serum content are upregulated in fatty liver disease [9, 10, 32].

## Dietary influences on hepatic lipogenesis

DNL rates will increase in the fed state with a macronutrient-balanced eucaloric diet. Increasing the percentage intake of carbohydrate will, however, dramatically augment both fasted and fed fractional lipogenesis (reviewed in [17]). As discussed in more detail below, fructose-sweeting beverages are associated with increased liver fat and fractional lipogenesis rates [33, 34], and as a result have been implicated as a contributing factor to NAFLD development [35]. On the other hand, a high starch meal reduces DNL rates [18]. Moreover, diets high in saturated long-chain fatty acids seem to have little impact on hepatic DNL [36]. Furthermore, fish oil supplementation which can lower fructose-induced hypertriglyceridemia, also showed no effect on DNL rates [37]. There are conflicting animal results regarding the role of medium-chain fatty acids on FASN activity and DNL [38–40], making interpretation difficult. Further, there is little research done to date how the consumption of medium- or short-chain lipids influences hepatic DNL in humans. In addition to macronutrient content, another factor effecting lipogenic response may be meal composition itself. Some studies [22, 41], but not all [42] have shown an effect of meal administration on DNL. Oral administration of a test meal results in greater lipogenic rate than duodenal feeding tube, due to small insulin response and slow nutrient delivery associated with the latter [22, 41]. Furthermore, placement of a feeding tube may elicit stress that can dramatically influence fatty acid fluxes. Overall, attention is required to ensure subjects maintain their normal diet or a eucaloric diet prior to study participation and any test meals should be orally consumed where applicable.

## Fructose is a lipogenic stimulant

Since DNL is upregulated in the fed state, administering a test meal can drive fatty acid synthesis rates. A carbohydrate-rich meal (80 % carbohydrate) given as a single bolus can increase DNL rates 3-times over fasting values [21]. Moreover, the type of carbohydrate supplied will influence the net increase. When glucose alone was compared to 50:50 or 25:75 glucose/fructose liquid meals, the presence of fructose significantly augmented DNL rates [43, 44]. Furthermore, fructose-induced lipogenic response increases in a dose-dependent manner [44]. This is a noteworthy finding since fructose overfeeding is strongly associated with fatty liver disease [33, 34]. In addition, Stanhope et al. found that after 10 weeks of fructose or glucose sweetened-beverage intake, DNL increased from 11 % to 17 % with fructose consumption only [45]. Moreover, a high fructose diet increased fasting DNL from 2 % to 9 % [37]. Fructose preferentially enhances hepatic lipogenesis over glucose since it initially metabolized by fructokinase which is not rate limiting and subsequently broken-down into 3-carbon molecules used for TG synthesis (Fig. 2), and is a potent stimulator of transcription factors regulating lipogenesis (reviewed in [34, 35]). Additionally, fructose feeding results in smaller glycemic excursions and lower insulin response compared to glucose [46], driving substrates towards lipogenesis. The mechanisms of fructose-induced DNL have been thoroughly describes elsewhere [33, 35]. From a clinical perspective, employing fructose as a challenge agent for DNL assessment is a potent stimulator of fatty acid synthesis, examining the system’s maximum capacity. Furthermore, fructose administration during tracer studies is an effective manner to evaluate the efficacy of a therapeutic intervention on liver fat metabolism [47].

## Measuring hepatic DNL in clinical research

Accessing liver biopsy material for DNL assessment can be risky and difficult, therefore a number of alternative non-invasive approaches have been developed to gain insight into fatty acid synthesis rates in humans. In a clinical setting, DNL can be determined by stable isotope tracer studies, fatty acid profiling or indirect calorimetry; the merits and limitations of each technique are described in Table 1.

**Table 1:** Advantages and disadvantages of clinical DNL techniques

DNL Technique	Advantages	Disadvantages
Stable label isotope	Fluxed based assay Selection of stable isotopes Moderate sensitivity Reproducible results	Cost of stable label substrates Enrichment period (up to 7 days prior to experiment) Requires specialized equipment for analysis
Fatty acid profiling	Relatively inexpensive Simple to execute High sensitivity High throughput	Requires specialized equipment for analysis
Indirect calorimetry	Non-invasive (no blood sample required)	Not hepatic specific

## Stable isotope tracer studies

Stable isotope tracers are safe for human use and are widely employed to examine lipid fluxes, turnover rates, lipoprotein production and secretion as well as DNL [48]. In addition, this technique can be readily combined with other measures of metabolism such as clamps, indirect calorimetry or MRS measurements [21, 49]. Moreover, stable isotope DNL measurements are highly reproducible [43]. Newly synthesized fatty acids, as well as those coming from the diet are packaged into TG and secreted from the liver as very low density lipoprotein (VLDL)-TG (Fig. 2). A recent study by Poulsen et al. eloquently demonstrated increased VLDL-TG secretion rates in NAFLD men under fasting as well as insulin stimulated conditions [50]. Hepatic DNL rates can be determined by tracking the VLDL-TG fraction following administration of a labeled substrate. VLDL-TG serves as a marker for assessing the hepatic TG pool. Serum lipoprotein fractions are isolated through ultracentrifugation. Some studies examine DNL rates in the TG-rich lipoproteins (TRL) fraction [43, 46], which is typically identified by a Svedberg flotation rate (Sf) of 60–400, however this fraction may contain both liver and intestinal DNL lipids, therefore it does not reflect a purely hepatic rate. The lipoprotein fraction can be further identified through immunoaffinity chromatograph by apolipoprotein detection for the VLDL-TG fraction. For analysis, lipids are then converted to fatty acid methyl-esters (FAME) before processing via gas chromatograph mass spectroscopy (GC-MS). Moreover, while most studies measure label-incorporation into palmitic acid (16:0), this is not a true estimate of total %DNL as oleic (18:1n9) and myristic (14:0) acids are also major fatty acids produced by FASN [26]. Therefore, lipoprotein fraction (TRL vs. VLDL-TG) and lipid species measured are key methodological considerations that may influence data interpretation and results.

## Mass Isotopomer Distribution Analysis (MIDA)

To determine biosynthesis rates, the enrichment or specific activity of the precursor substrate is required. Since obtaining biopsy material is risky, not always feasible, and is subjected to collection site bias; MIDA was developed to overcome these issues. MIDA estimates the amount of stable isotope in a given tissue like the liver; this analysis was first introduced and applied to lipogenesis studies by Hellerstein and Neese [51]. It is based on the combinatorial probability of polymers repeating from a monomer. The local enriched precursor (e.g. acetyl-CoA) is determined by the relative distribution pattern of labeled isotopomer in the product (e.g. VLDL-palmitate). In the case of palmitate, the isotopomers could contain anywhere from zero to eight labeled monomers. The fractional abundance of the unlabeled end-product (*M*_*0*_), and those moieties containing one (*M*_*1*_), two (*M*_*2*_) or more (*M*_*n*_) labeled acetate subunits are measured at baseline and in enriched states, then the fractional rate of lipogenesis is calculated from the rate at which enrichment increases in the product. Before MIDA, errors in estimating biosynthesis rates were unavoidable due to the uncertainty of cytosolic dilution of the precursor substrate [52].

## Selecting a stable isotope substrate

Typically either stable labeled ^13^C-acetate or deuterated water (^2^H_2_O) are employed for the DNL isotope technique, and the ideal substrate chosen for a particular study depends on the method of administration (iv. infusion vs. consumption), length of isotope priming, and complexity of the study, for example if multiple stable isotopes are used. These considerations are addressed below.

### ^13^C-acetate

This tracer is a commonly used substrate in current DNL studies. In the body, acetate is converted to acetyl-CoA which is the precursor substrate of fatty acid synthesis. Infusion with either [1-^13^C]-acetate or [2-^13^C]-acetate show similar results [42], however many researchers have adopted [1-^13^C]-acetate as a standard precursor. ^13^C-acetate administration does require a lengthy intravenous infusion time prior to testing. Studies have shown that increasing the infusion time results in a greater %DNL rate; typically fractional rates are <5 % with a short infusion of 8 hours and increases to >3-fold after a 24- h dose in a similar cohort [18, 41]. As a result, the increase of acetate tracer into the precursor pool is in large quantities (several grams). However, longer infusion times are required to overcome a delay in VLDL-TG appearance due to entry into a storage pool [23]. Although extremely rare, there exists a possibility that an acetate precursor may have two labeled carbons – one in the defined position (for example 1-C), and the other carbon labeled occurring due to natural abundance, which would thus be termed *M*_*2*_. When combined with other acetates in a fatty acid chain the frequency of *M*_*2*_ and *M*_*3*_ lipids would increase and the occurrence of *M*_*0*_ and *M*_*1*_ would be reduced, affecting the calculated rate. However, this is solved by expressing the excess isotopomeric distribution (*EM*_*2*_/*EM*_*1*_; natural abundance corrected) rather than the absolute isotopomer frequencies *M*_*2*_/*M*_*1*_ [42].

### Deuterated water

The labeled hydrogen atoms in deuterated water (^2^H_2_O) can be incorporated into the fatty acid acyl chain through several means; through a number of water requiring pathways, such as glycolysis and TCA cycle, by direct hydrogen-exchange or, obtained from NADPH as a reducing equivalent for FASN. Furthermore, the proportion of NADPH hydrogen atoms deriving from water will depend on the route of NADPH formation; for example via the pentose pathway, malic enzyme or other enzymatic routes. While earlier studies dosed deuterated water for 48-h prior to testing resulting in low DNL rates (reviewed in [53]), more recent studies have administered heavy water up to 1 week prior to the investigation test day allowing for maximal capture of DNL rates. Typically, a diluted (70 % ^2^H_2_O) priming dose is administered followed by several maintenance doses [25–27, 49]. Administration of the label is given orally which is an advantage over the stress that may be associated with lengthy ^13^C-acetate infusion studies. Also, the isotope is relatively inexpensive. Deuterated water is also a preferred stable isotope substrate when other ^13^C-labels substrates are being used, or if the study procedures do not accommodate an IV infusion. On the other hand, there are several shortcomings associated with deuterated water DNL-measurements. In early research, administration of deuterated water induced vertigo [54]. However, improvements to mass spectroscopy instrument sensitivity have prevented the need of heavy water doses large enough to provoke this side-effect. Other limitations include the assumption that plasma ^2^H enrichment is the same as tissue intracellular pools, moreover the ratio of ^2^H:H may not be consistent over various metabolic conditions [55]. In addition, an isotope bias may also exist influencing %DNL rates. Deuterated-DNL rates were originally reported as parts per thousand change in sample enrichment relative to natural abundance (standard mean ocean water), and used literature based hydrogen/carbon incorporation ratios to estimate synthesis rates [55], which are impacted by several errors and assumptions [56]. However, a variation of MIDA has been utilized for determining the %DNL with deuterated water as a precursor [57], reconciling previous limitations.

### ^13^C-glucose and ^13^C-fructose

Labeled fructose and glucose have been examined as potential DNL tracers however, these are not consider suitable substrates to determine fatty acid synthesis rates. Chong and colleagues demonstrated, approximately 38 % of the label was identified in TG-glycerol fraction, and only minimal amounts (~0.5 %) were found within TG-fatty acids [46]. Although administration these labels most closely resembles real life situation such as the consumption of a sweetened beverage, this method does not convey the total %DNL since the majority of the labeled sugars were found to be oxidized [46].

### ^13^C-ethanol

Excessive alcohol consumption is often associated with hepatosteatosis, and even acute alcohol intake can upregulate fractional DNL up to 30 % [58]. Since ethanol is converted to acetate (a substrate for FASN) by acetyl-CoA synthetase, ^13^C-ethanol was examined as a potential tracer for DNL assays. Siler et al. found that approximately 70-80 % of the labeled ethanol was found in plasma as enriched acetate, yet <5 % of the label was identified in VLDL-palmitate [58]. Therefore, while alcohol stimulates hepatic lipid accretion, this is not a direct effect through the provision of acetate-loading in an acute setting and therefore not ideal for experimental purposes. Furthermore, during a clinical study, subjects should be instructed to avoid alcoholic beverage prior to testing due to its stimulatory effect on the DNL pathway.

## Fatty acid profiling

**Fig. 4:**
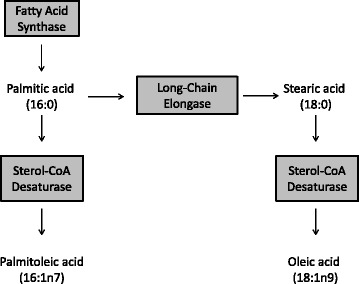
*De novo* fatty acids. The saturated fatty acid palmitic acid is the main product of fatty acid synthase. Long-chain elongase (LCE) can extend palmitic acid by 2-carbons to generate stearic acid. Palmitic and stearic acids serve as precursors for palmitoleic and oleic fatty acids respectively, where a double bond is incorporated into the fatty acid chain by sterol-CoA desaturase (SCD)

Lipidomics is the study of lipid species and their biological roles, and this emerging field has advanced medical research. Often lipids are altered in a diseased state and novel lipid biomarkers can be useful for diagnosis and evaluating progression and treatment response to a disease [59]. Improvements in sample preparation resulting in greater recovery and efficiency as well as the ability to detect low abundance lipids have significantly contributed to this growing field. For blood samples, mass spectrometry (MS) coupled with chromatography separation is the most widely used technique for lipid quantification, due to the high sensitivity and throughput of this method. Either liquid chromatography (LC) or GC can be integrated for lipidomic studies, however GC-MS is preferred for fatty acid profiling since it is a more rapid and sensitive method. GC tends to yield higher peak resolution than LC. In this respect, fatty acid profiling and lipid indexes are a highly sensitive, alternative approach to tracer studies to examine DNL in a clinical setting (reviewed in [33]). The synthesis of these *de novo* fatty acids is shown in Fig. 4.

### Profiling de novo fatty acids

Increased DNL has been described as a key player in the development of hepatic lipid accretion and NAFLD. This was elegantly shown by Puri et al., through fatty acid profiling in both plasma and liver biopsy samples from NAFLD patients [60, 61]. NAFLD subjects demonstrated an increase in *de novo* lipid species composition, as well as relative and absolute amounts of fatty acids such as palmitic, palmitoleic (16:1n7), stearic (18:0) and oleic (18:1n9) acid. In support of these findings, Lee et al. observed elevated plasma palmitoleic acid (16:1n7) levels in MRS-confirmed fatty liver subjects compared to low fat liver controls [62]. In addition, palmitoleic acid (16:1n7) levels significantly correlated with isotope DNL assessments. More recently, hepatic oleic acid (18:1n9) concentrations determined by ^1^H-NMR spectroscopy was found to significantly associate with histology quantified steatosis in NAFLD patients undergoing bariatric surgery [63].

### Fatty acid indexes

In animal models [64] as well as human subjects [60, 61, 63], hepatic steatosis is consistently observed with an attenuation of the polyunsaturated to monounsaturated fatty acids ratio, therefore a number of lipid ratios and indexes have been developed as a surrogate marker of DNL. The lipogenic index introduced by Hudgins et al. in 1996, calculates the ratio of palmitic acid (16:0) to the essential omega-6 linoleic acid (18:2n6), reflecting DNL rates when dietary fats were matched for adipose tissue fatty acid composition [65]. More recently, Chong et al. used this method to evaluate lipogenesis after the consumption of a 3-day high carbohydrate diet [66]. Indeed, the lipogenic index was higher after high carbohydrate feeding compared to a high fat diet. This index has been also analyzed in erythrocyte membranes in the EPIC-Potsdam Study and found to positively correlate with proxies of liver fat content [67]. Other DNL indices include the desaturation index, measuring the ratio of oleic acid (18:1n9) to stearic acid (18:0). Although tested in dogs, this index did not change over an 8-h postprandial phase following a high sucrose meal [68]. However when comparing the TG and phospholipid desaturation index, Peter et al. did find an association with liver fat content in humans with NAFLD [69]. Finally, examining the percent increase in VLDL-palmitate may be a significant biomarker of DNL, as the percent change strongly correlated with the change in %DNL fractional rate determined by MIDA over an 8-h postprandial period in response to a fructose challenge [44]. Taken altogether, fatty acid indexes are an effective method for DNL examination and may be considered a suitable alternative to tracer studies. However due to the limited number of studies performed to date in humans; caution is advised when applying this measurement to evaluate the effect of DNL in response to a therapeutic treatment.

## Indirect calorimetry

The DNL reaction is energy demanding, requiring 14 NADPH resulting in the production of 7 CO_2_ for the generation of 1 palmitate fatty acid. In this respect, indirect calorimetry, a powerful technique used to examine substrate utilization and energy expenditure, has been also widely used as a method to determine whole-body DNL in vivo. Through gas exchange, the respiratory quotient (RQ); the ratio of carbon dioxide production to oxygen consumption, can be calculated. RQ > 1.0 indicates a net conversion of carbohydrate to lipid. One caveat of this measurement is that it cannot ascertain hepatic-specific DNL *per se*, as only net whole-body DNL is measured, however, it has been suggested that short-term, postprandial values of RQ > 1.0 would likely reflect a prominent hepatic contribution [21]. Moreover, this technique may be better suited for some groups over others. For instance, after a carbohydrate-rich meal, net lipogenesis was significantly greater in obese men compared to lean controls [21], however in overweight men and women this trend was not observed [70]. On the other hand, several researchers have found that the RQ does not reach over the lipogenesis threshold when examining carbohydrate-induced DNL in healthy lean populations, as excess dietary carbohydrates were shunted towards glycogen storage [19, 71]. Only after glycogen storage capacity is saturated, increases in DNL can be observed in normal healthy populations through indirect calorimetry. In general, this can be achieved with an extreme dietary intervention such as massive carbohydrate overfeeding, increasing glycogen by ~500 g [72]. Therefore, as a complementary tool, indirect calorimetry can provide valuable clinical information regarding whole-body metabolism such as fuel selection and energy expenditure which would ultimately impact liver function, however there are many shortcomings associated with using this technique solely for hepatic DNL determinations.

## The role of DNL assessment in evaluating NAFLD treatment

There is currently no NAFLD/NASH-specific medication available on the market. The unmet needs of those with this metabolic disorder have been addressed with lifestyle modifications for weight loss, vitamin supplements [2, 73] or insulin sensitizing therapy such as pioglitazone treatment [74]. While these therapeutic agents have yielded some success [75], concerns of increased cardiovascular risks [76] and undesired side-effects [77–79] remain. Interestingly, pioglitazone treatment, but not rosiglitazone, significantly lowered steady-state fractional DNL in type 2 diabetes subjects with hyperglycemia [80], demonstrating one of pioglitazone’s mechanisms as an effective anti-NAFLD agent. In addition to a number of anti-fibrotic and anti-inflammation products under development, drug classes with steatosis lowering capacity are being rigorously investigated for NAFLD/NASH treatment. However, to our knowledge, only three other published studies to date have evaluated stable-label isotope DNL response as mode of drug efficacy in the context of fatty liver disease (Table 2). A novel ACC inhibitor (identified as compound 9) dramatically lowered peak fructose-induced fractional DNL by 64 % compared to placebo after a single dose in a phase 1 clinical study [47]. Moreover, the GLP-1 agonist Liragultide reduced change from baseline %DNL compared to placebo in NASH patients [29]. On the other hand, colesevelam, a bile acid sequestrant, potent cholesterol-lowering agent and putative anti-NASH agent, showed no effect on hepatic DNL [81]. In addition to pharmacological interventions, a number of nutraceutical products have been shown to display anti-dyslipidemia properties (reviewed in [82]) and even have been evaluated as anti-NASH agents [83]. Regarding hepatic DNL, phytochemicals from mulberry leaf and chokeberries as well as other plant sterols have shown promising pre-clinical lowering effects in animal models of NAFLD [84–86] however further studies in humans are needed to evaluate their effectiveness.

**Table 2:** Summary of NAFLD/NASH clinical trials incorporating stable-label isotope DNL assessments

Subjects	Study Type	Intervention	Period	Tracer	Outcome	Ref
Healthy controls (*n* = NR)	Randomized double-blind, placebo-controlled crossover	Compound 9 (600 mg) or placebo with one week washout	Single dose	[1-^13^C]-acetate (9–9.5 mg/min) for 20.5 h	Compound 9 reduced fructose-induced fractional DNL	[47]
Type 2 diabetes (*n* = 12)	Open label, randomized (1:1)	Pioglitazone (dose escalated to 45 mg/day) Rosiglitazone (dose escalated to 4 mg twice daily)	20 weeks	[1-^13^C]-acetate (10 mg/min) For 12 h	Pioglitazone reduced fasting fractional DNL over course of acetate infusion No change with Rosiglitazone	[74]
Type 2 diabetes (*n* = 60)	Randomized double-blind placebo-controlled (1:1)	Colesevelam (3.75 g/day) or placebo	12 weeks	[1-^13^C]-acetate (10 mg/min) 19.5 h continuous infusion	Placebo increased fasting and postprandial fractional DNL, no change with colesevelam	[81]
Biopsy-proven NASH (*n* = 14)	Randomized double-blind, placebo-controlled (1;1)	Liraglutide (dose escalated to 1.8 mg/day) or placebo	12 weeks	^2^H_2_O (3 g/kg total body water in 2 doses) plus *ad libitum* drinking water enriched with 0.4 % ^2^H_2_O over 20 h	Liraglutide decreased fasting % change DNL	[29]

*NR* Not reported

## Conclusions

Upregulated DNL is a contributing factor to hepatic steatosis and NAFLD [20, 27, 61]. There are several methodologies to evaluate DNL in a clinical setting. Currently, only a handful of laboratories have the capability and expertise to examine fractional DNL rates with stable isotopes. However, as pharmaceutical and biotech companies develop novel anti-NAFLD drugs that target components of steatosis development such hepatic DNL, it is anticipated that more studies will evaluate DNL through the stable label fractional technique. On the other hand, fatty acid profiling may represent an attractive alternative. There are several limitations associated with indirect calorimetry to measure DNL, however as complementary analysis, this measurement can provide insight into hepatic and whole-body metabolism. In summary, measurements of hepatic lipid accumulation such as DNL assessment either by stable label technique or fatty acid profiling, will become an instrumental marker of drug efficacy for novel NAFLD investigational products and nutraceutical treatment response.

## Abbreviations

ACC: Acetyl-CoA carboxylase
ACP: Acyl-carrier protein
CPT1: Carnitine palmitoyl transferase 1
DNL: *De novo* lipogenesis
FASN: Fatty acid synthase
FDA: Food and Drug Administration
GC: Gas chromatography
LC: Liquid chromatography
MIDA: Mass isotopomer distribution analysis
MRS: Magnetic resonance spectroscopy
MS: Mass spectrometry
NAFLD: Non-alcoholic fatty liver disease
NASH: Non-alcoholic hepatosteatosis
RQ: Respiratory quotient
TG: Triglycerides
TRL: TG-rich lipoproteins
TZD: Thiazolidinediones
VLDL: Very low density lipoprotein
